# Authors’ Reply

**Published:** 2010

**Authors:** Sachin Gupta, Deepak Govil, Prem N. Kakar, Om Prakash, Deep Arora, Shibani Das, Pradeep Govil, Ashima Malhotra

**Affiliations:** Department of Anaesthesiology, Max Super Speciality Hospital, 1Press Enclave road, Saket, New Delhi, India

I appreciate that you went through the article.[1,2] Just wanted to add a few points in reply.

Apart from the organisms mentioned, colistin is inherently resistant to *Brucella* and *Burkholderia* cepacia *species. Salmonella, Shigella* and several mycobacterial species require higher minimum inhibitory concentration (MIC) to attain effect.

Aerosolization of colistin is now becoming a standard adjuvant therapy for Ventilator Associated Pneumonia. It should be given through ultrasonic nebulizers to reduce the wastage of the drug and to achieve maximum concentration in the lungs. Bronchospasm is less common than it is thought to be but it should be followed by bronchodilator nebulization to prevent this side effect.[3] No data are available for dose adjustment for nebulization in renal disease, so it is still a debatable issue.

Similarly, neurological side effects are more theoretical. Data from the recent literature suggest that the use of colistin is associated with lower and less severe toxicity compared to that reported in the old literature. No episodes of neuromuscular blockade or apnea induced by polymyxins have been reported in the literature over the past 15 years or more.[4]

As far as superinfection is concerned, it can happen with use of any broad-spectrum antibiotic like carbepenems for prolonged duration or with polypharmacy and is not specific for colistin.
